# Study Protocol for a Stepped-Wedge Cluster (Nested) Randomized Controlled Trial of Antenatal Colostrum Expression (ACE) Instruction in First-Time Mothers: The ACE Study

**DOI:** 10.1177/08903344231215074

**Published:** 2023-12-29

**Authors:** Cassandra Cuffe, Roslyn Giglia, Matthew N. Cooper, Julie Hill, Desiree Silva, Anita M. Moorhead, Valerie Verhasselt, Joshua R. Lewis, Deborah Ireson, Jill R. Demirci, Talea Cotte, Kathryn Webb, Frances Patey, Therese A. O’Sullivan

**Affiliations:** 1 Nutrition & Health Innovation Research Institute, School of Medical and Health Sciences, Edith Cowan University, Joondalup, WA, Australia; 2 Adjunct Academic, Curtin University Kent St, Bentley WA, Australia; 3 Telethon Kids Institute, University of Western Australia, Perth Children’s Hospital, Perth, WA, Australia; 4 Telethon Kids Institute, Perth, WA, Australia; 5 School of Medicine, The University of Western Australia, Perth, WA, Australia; 6 School of Medical and Health Sciences, Edith Cowan University, Perth, WA, Australia; 7 La Trobe University, Judith Lumley Centre, Melbourne, VIC, Australia; 8 The Royal Women’s Hospital, Parkville, VIC, Australia; 9 Larsson-Rosenquist Foundation Centre for Immunology and Breastfeeding, School of Medicine, University of Western Australia, Perth, WA, Australia; 10 Medical School, The University of Western Australia, Perth, WA, Australia; 11 Centre for Kidney Research, Children’s Hospital at Westmead, School of Public Health, Sydney Medical School, The University of Sydney, Sydney, NSW, Australia; 12 School of Nursing and Midwifery, Edith Cowan University, Bunbury, WA, Australia; 13 Department of Health Promotion & Development, School of Nursing, University of Pittsburgh, Pittsburgh, PA, USA; 14 Armadale Kalamunda Group, Armadale Hospital, Perth, WA, Australia

**Keywords:** antenatal colostrum expression, breastfeeding, formula feeding, human milk, human-milk substitute, pregnancy, randomized controlled trial, secretory activation, supplementation, The ORIGINS Project

## Abstract

**Background:**

Although many mothers initiate breastfeeding, supplementation with human-milk substitutes (formula) during the birth hospitalization is common and has been associated with early breastfeeding cessation. Colostrum hand expressed in the last few weeks before birth, known as antenatal colostrum expression (ACE), can be used instead of human-milk substitutes. However, evidence is lacking on the efficacy of ACE on breastfeeding outcomes and in non-diabetic mothers.

**Methods and Planned Analysis:**

This multicenter stepped-wedge cluster (nested) randomized controlled trial aims to recruit 945 nulliparous pregnant individuals. The trial is conducted in two phases. During Phase 1, control group participants are under standard care. During Phase 2, participants are randomized to ACE instruction via a pre-recorded online video or a one-on-one session with a midwife. Adjusted logistic regression analysis will be used to examine the relationship between ACE instruction and breastfeeding outcomes.

**Research Aims and Questions:**

Primary aim: (1) Does advising pregnant individuals to practice ACE and providing instruction improve exclusive breastfeeding rates at 4 months postpartum? Secondary research questions: (2) Do individuals who practice ACE have higher rates of exclusive breastfeeding during the initial hospital stay after birth? (3) Is teaching ACE via an online video non-inferior to one-on-one instruction from a midwife? (4) Does expressing colostrum in pregnancy influence time to secretory activation, or (5) result in any differences in the composition of postnatal colostrum?

**Discussion:**

Trial findings have important implications for maternity practice, with the online video providing an easily accessible opportunity for ACE education as part of standard antenatal care.

**Key Messages**

- Evidence is lacking on the effectiveness of antenatal colostrum expression on breastfeeding outcomes and in non-diabetic mothers.
- This stepped-wedge cluster (nested) randomized controlled trial is being conducted at multiple hospital sites in Western Australia and recruits first-time mothers.
- To our knowledge, this is the first large scale study being conducted in the non-diabetic population of pregnant individuals that will determine the effectiveness of various formats of antenatal colostrum expression education on breastfeeding outcomes.

## Background

Exclusive breastfeeding is when the infant receives only human milk, except for oral medicines, vitamins, and minerals, and it is recommended for the first 6 months of life (World Health Organization [WHO] & the United Nations Children’s Fund [UNICEF], 2021). Supplementation with human milk substitutes (like commercial infant formula) during the initial hospital stay after birth is common in Australia, with approximately one-third of infants receiving human milk substitutes during the birth hospitalization (Netting et al., 2022). Supplementation with human milk substitutes jeopardizes exclusive breastfeeding practices and increases the risk of early breastfeeding cessation (McCoy & Heggie, 2020; Vehling et al., 2018). As an alternative to the use of human milk substitutes, colostrum can be expressed in pregnancy, stored in a freezer, and taken to the hospital at the time of birth. Colostrum expressed in pregnancy can be defrosted and fed to infants during the initial hospital stay if additional supplementation is required. Supplementation with a parent’s own colostrum instead of human milk supplements may protect exclusive breastfeeding practices in hospitals.

Historically, there have been concerns that antenatal colostrum expression (ACE) can affect hormones that promote the onset of labor (e.g., oxytocin). To date, there has been only one large-scale and high-quality randomized controlled trial (RCT) of ACE conducted among pregnant individuals with low-risk diabetes (the Diabetes and Antenatal Milk Expressing [DAME] study). The DAME trial demonstrated that ACE twice per day for up to 10 min beginning at 36 weeks gestation was safe for pregnant individuals with diabetes who were otherwise at low risk of pregnancy or general health complications. In the DAME trial, there was no difference observed between randomization groups for admissions to the Neonatal Intensive Care Unit (NICU) or mean gestational age at birth (Forster et al., 2017).

A scoping review by Foudil-Bey et al. (2021) evaluated ACE outcomes and concluded that there was an increasing interest in the safety, efficacy, and acceptability of ACE. Specific to this protocol, Foudil-Bey et al. (2021) found that of the 20 studies included, breastfeeding success was not clearly defined, and the duration of follow-up varied greatly between studies. Similarly, the implementation of ACE across maternity hospitals or other prenatal care settings is unclear. Across the United Kingdom, the National Health Service (NHS) guidelines at major maternity centers advise that any pregnant individual can practice ACE from 36 weeks gestation onward, but it is promoted as being particularly useful for mothers at risk of low milk supply or if the infant has an increased risk of being born with hypoglycemia. The NHS states that other individuals who may benefit from ACE are those planning an elective Caesarean birth or with a body mass index (BMI) > 35 kg/m^2^. Some health districts across Australia and New Zealand encourage ACE for pregnant individuals with medical conditions that might cause the infant to have hypoglycemia at birth, or potentially if the mother has had previous breastfeeding difficulties. Information brochures are accessible online detailing how to hand express and store colostrum in pregnancy and describe situations where ACE may be beneficial (i.e., gestational diabetes mellitus). Currently, ACE is not routinely or universally promoted for all eligible pregnant individuals entering maternal health care. This is likely due to a paucity of evidence regarding the efficacy of ACE in improving breastfeeding rates in the general pregnant population and a lack of safety data for ACE in non-diabetic populations that may otherwise be considered to have a high-risk pregnancy. Further, little research has been carried out on the optimal delivery methods for information and instruction related to ACE.

Some qualitative research suggests ACE may shorten the time until a mother experiences onset of copious human milk (secretory activation, previously known as Lactogenesis II; Demirci et al., 2019); however, there is currently no high-quality evidence to support this claim. ACE has the potential to improve breastfeeding outcomes for all pregnant individuals, not just those with health conditions such as diabetes, or with previous breastfeeding difficulties. Results of this trial will inform standard antenatal care processes for the general population of pregnant individuals. In addition, the analysis of any effect(s) that ACE may have on postnatal colostrum composition is important, given the vast health benefits of colostrum for infants.

The trial has been designed to answer the following research questions:

1. (Primary aim) Does advising pregnant individuals to practice ACE and providing instruction improve exclusive breastfeeding rates at 4 months postpartum, compared to standard care? The hypothesis is that pregnant individuals who receive ACE education will have higher rates of exclusive breastfeeding at 4 months (EBF4M) compared to pregnant individuals in the control group receiving standard care.
2. Do pregnant individuals who receive ACE instruction have lower rates of humanmilk substitute (formula) use during the initial hospital stay after birth compared to mothers who do not receive ACE instruction? The hypothesis is that mothers who receive ACE instruction will have lower rates of humanmilk substitute use during the initial hospital stay.
3. Is teaching ACE via an online video non-inferior to one-on-one instruction from a midwife [in terms of breastfeeding outcomes]? The hypothesis is that teaching ACE via an online video is non-inferior to one-on-one instruction from a midwife.
4. Does expressing colostrum in pregnancy influence time to secretory activation, compared to not expressing colostrum in pregnancy? The hypothesis is there will be no difference in the time it takes until secretory activation occurs between individuals who practice ACE and individuals who do not practice ACE.
5. Does expressing colostrum in pregnancy result in any differences in the composition of postnatal colostrum? The hypothesis is that there will be no differences in post-birth colostrum content in macronutrients and key bioactive compounds—Immunoglobulin A (IgA), Transforming Growth Factor (TGF)-beta 1, soluble CD14, Lactoferrin and Epidermal Growth Factor (EGF)—between individuals who express colostrum antenatally, compared to individuals who do not.

## Methods

### Research Design

The ACE Study is a stepped-wedge cluster (nested) randomized controlled, investigator-blinded, multicenter, non-inferiority trial with parallel intervention groups. The rationale for this trial design (Phase 1, a period where the control group data will be collected, and Phase 2, randomization of participants to either the midwife [MW] or video instruction [VI] groups) was chosen as it provides a quarantined period of observation and data collection from the control group. Randomizing participants to all three groups simultaneously was not performed as this would risk contamination (via artificially increasing knowledge above standard care) in the control group and hospital staff delivering care. Pregnant individuals attending the same antenatal classes may discuss and share aspects of their care, and hospital staff may inadvertently promote ACE to the control group. Each hospital (site) is a cluster, with the Phase 1 start time being dependent on hospital logistics (for example ability to carry out staff training, ethics, and governance approvals). During Phase 2, nested within each cluster, individual participants are randomized (50:50) to one of two invention arms. The trial has a primary endpoint of 4 months postpartum, specifically the rate of EBF4M. All enrolled participants will have data collected until 4 months postpartum. The endpoint in this trial was chosen as a timepoint where the achievement of the proposed level of breastfeeding would be significant, given previous national indicators (Australian Bureau of Statistics, 2017–2018).

#### Choice of comparators

Participants in the non-intervention (control) group will receive standard maternity care. Participants individually randomized to one of two intervention arms will receive the same ACE instruction from either a one-on-one session with a midwife (MW group) or ACE instruction from an online video (VI group). Participants in the MW group have a 15 to 20-minute one-on-one consultation with a midwife (also trained as an International Board-Certified Lactation Consultant [IBCLC]) who provides information and instruction on how to hand express, collect, and store colostrum in pregnancy. The midwives providing the ACE instruction are trained in the study protocol for teaching hand expression in pregnancy. Participants randomized to this group can ask questions and practice the technique directly with the midwife. Participants randomized to the VI group receive a link to an online 16-minute video they can watch at home as many times as they like. The ACE video used in this trial was developed by members of the ACE Study research team to determine whether an online instructional video could improve pregnant individuals’ knowledge and confidence around breastfeeding. The video was made specifically for pregnant individuals and provides education and instruction on how to hand-express, collect and store colostrum during pregnancy. In a previous evaluation of the video, 95 pregnant individuals completed questionnaires before watching the video, and again afterwards; researchers found the video improved knowledge scores and self-reported confidence around ACE (O’Sullivan et al., 2019).

#### Ethics

This trial is being conducted according to the strict protocols of the National Health and Medical Research Council (NHMRC) National Statement on Ethical Conduct in Human Research (NHMRC et al., 2007). All procedures involving human participants/patients were approved by the Human Research Ethics Committee (HREC) at Edith Cowan University (ECU) on July 8, 2019; the HREC approval number is 2019-00572-OSULLIVAN. The ACE Study has been approved by Ramsay Health Care Western Australia and South Australia, HREC (reference number: 1901), and Women and Newborn Health Service Ethics Committee (RGS0000001504). The ACE Study has been registered as a clinical trial with the Australian New Zealand Clinical Trials Registry (Universal Trial Number: U1111-1232-1397, https://anzctr.org.au/Trial/Registration/TrialReview.aspx?id=377448).

#### Study framework

**Figure 1.**
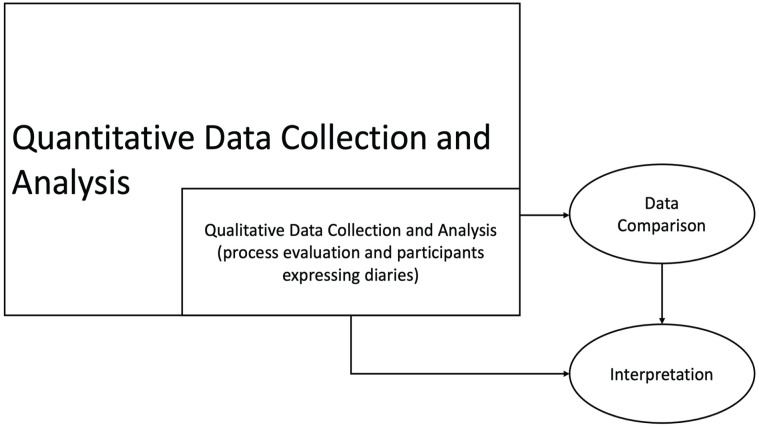
Theoretical Framework for the ACE Study. *Note*. ACE = antenatal colostrum expression.

The ACE Study draws on the pragmatist paradigm and uses a mixed methods model with an embedded experimental design to collect quantitative and qualitative data (Creswell, 2014). A philosophical movement that originated in the 1870s, pragmatism originated when a group of scholars rejected traditional ideas and assumptions about the nature of reality, knowledge, and inquiry (Kaushik & Walsh, 2019). Pragmatism is based on the assumption that researchers use philosophical and methodological approaches that are best suited to their research problem (Kaushik & Walsh, 2019). One of the major underpinnings of a pragmatist paradigm is that a person’s perceptions of the world, or their knowledge, are influenced by their social experiences (Kaushik & Walsh, 2019). In the ACE study, quantitative data collection is given priority and guides the study while the qualitative method of data collection is nested within the larger study design (Alavi et al., 2018; Figure 1). Quantitative and qualitative data from the ACE study will be analyzed separately; however, the findings from the qualitative data will be used to understand the participants’ views within the context of the experimental intervention (Creswell, 2014).

### Setting and Relevant Content

Western Australia (WA) accounts for 33% of the Australian continent and 10% of the country’s population. Perth is the capital city and home to 75% of the state’s population. It stretches along the western coastal border to the north and south of the city. Proposed recruitment sites include Joondalup Health Campus Hospital (JHC), WA; Perth Pregnancy Centre, WA; Armadale Hospital, WA; Glengarry Private Hospital, WA; Fiona Stanley Hospital (FSH), WA; Bunbury Hospital, WA; and Busselton Hospital, WA. JHC is the largest healthcare facility in the northern suburbs of Perth. JHC is a metropolitan, private, and public hospital in a higher socioeconomic area. The Australian Bureau of Statistics (2016) Socio-Economic Indexes for Areas (SEIFA) ranks areas relative to socioeconomic advantage and disadvantage, using decile ranking (1–10). Decile 1 represents low socioeconomic communities with greater disadvantage and 10 represents greater socioeconomic advantage. JHC is located in an area with a decile score of 7. JHC includes participants recruited through the ORIGINS cohort as the ACE Study is a sub-project of The ORIGINS Project. JHC has approximately 4057 births per year, with a 39% Caesarean section rate (Hutchinson et al., 2019). Pregnant individuals receiving antenatal services at Perth Pregnancy Centre most commonly birth at JHC. Armadale Hospital is a metropolitan public hospital in a low socioeconomic area (decile score 1) with approximately 2513 births per year and a 23% Caesarean section rate (Hutchinson et al., 2019). Glengarry Private Hospital is in a high socioeconomic area (decile score 10) with approximately 593 births per year and a 49% Caesarean section rate (Hutchinson et al., 2019). FSH is a government hospital located in Murdoch, a high socioeconomic area (decile score 9), 15 km south of Perth’s central business district. FSH is the major tertiary hospital in the southern metropolitan area, with about 3600 births per year and a Caesarean section rate of 34% (Hutchinson et al., 2019). Bunbury (decile score 6) and Busselton (decile score 1) Hospitals are public hospitals in a regional area in Southwest, WA. There are approximately 1557 births per year in the South West Health Region and a 27% Caesarean section rate (Hutchinson et al., 2019).

#### Breastfeeding in Australia

In Australia, National Health Survey data shows that the prevalence of exclusive breastfeeding at 4 months of age is 61%, and drops to 29% by 6 months of age, with rates remaining consistent since 2014 to 2015 (Australian Bureau of Statistics, 2017–2018). The more recent National Health Survey data (2020 to 2021) was collected online during the COVID-19 pandemic and should be used for point-in-time analysis only and not be compared with previous survey data. The National Health Survey data for 2017 to 2018 came from a sample of approximately 21,300 people across Australia. Data was collected during personal interviews. One person was interviewed per household and provided breastfeeding data about one child in the household.

In Australia, those individuals not breastfeeding to 6 months are more likely to be primiparous, overweight or obese, have lower education, and smoke cigarettes (Moss et al., 2020). The primary reasons for not breastfeeding to 6 months in Australia include the perception of insufficient milk supply and other breastfeeding difficulties (e.g., latching issues, pain, expressing is too hard; Moss et al., 2020).

## Sample

The target population for this trial are nulliparous pregnant individuals aged 18 or older.

### Inclusion Criteria

Individuals are eligible if they have a singleton pregnancy, have adequate English language skills to provide informed consent, are willing to participate in the trial, are planning to give birth at one of the trial sites, and intend to breastfeed.

### Exclusion Criteria

The exclusion criteria are listed in Table 1. Individuals who develop any relevant maternal or infant medical conditions listed as exclusion criteria will not be eligible to participate/will be withdrawn from the trial.

**Table 1.** PICO (Population, Intervention, Comparison, Outcomes) for the ACE Study.

Study Component	Details
Population	Inclusion criteria
	● Nulliparous individuals
	● Singleton pregnancy
	● Able to read and speak in English
	● Planning to breastfeed
	● Willing to participate in the trial
	● Planning to give birth at one of the trial sites
	Exclusion criteria
	● EPDS score ≥13, or PASS score ≥26
	● Diabetes (pre-existing and pregnancy-induced)
	● Pre-eclampsia
	● Placenta previa
	● Antepartum hemorrhage
	● History of threatened/actual premature labor
	● Cervical incompetence
	● Fetal anomaly (cleft palate, down-syndrome, heart defect, neural tube defects)
	● Fetal compromise (polyhydramnios, known inter-uterine growth restriction)
	● Preterm birth (<37 weeks gestation)
Intervention	Intervention components in addition to standard maternity care during phase 2 include:
	● Oral and written information on antenatal colostrum expression: commence hand expressing from 37 weeks gestation, twice a day for 3-5 minutes per breast (maximum 10 minutes); and information on safe storage of colostrum during pregnancy
	● One-on-one ACE education session with midwife/IBCLC *or*
	● Unlimited access to an online 16-minute ACE instructional video
Comparison	Control group will receive standard maternity care
Outcomes	Primary outcome:
	● Exclusive breastfeeding at 4 months postpartum (EBF4M) [phase 1 control period versus phase 2 ACE groups (combined)]
	● Secondary outcomes:
	● Exclusive breastfeeding at 1, 4, 8, 12, and 16 weeks postpartum [MW intervention group versus the VI intervention group]
	● Exclusive breastfeeding from birth to discharge
	● Time to secretory activation
	● Colostrum composition after birth: macronutrients (lactose, protein, fat content), IgA, soluble CD14, Lactoferrin, EGF, and TGF
	Other outcomes:
	● Maternal attitudes to infant feeding
	● Breastfeeding self-efficacy
	● Time from starting ACE (obtained from milk-expressing diaries) until labor
	● Post-term induction rate
	● Gestational age at birth
	● Infant admission to special care nursery

*Note*. ACE = antenatal colostrum expression; EPDS = Edinburgh Perinatal/Postnatal Depression Scale; PASS = Perinatal Anxiety Screening Scale; IBCLC = International Board Certified Lactation Consultant; IgA = Immunoglobulin A; EGF = epidermal growth factor; TGF = transforming growth factor.

Pregnant individuals birthing at hospital trial sites who meet the inclusion criteria are approached to participate by either the midwife providing their antenatal care or an ACE Study researcher. Eligible individuals will be approached by the midwife between the 28- and 34-week antenatal clinic visits. The midwife conducts an eligibility screen to determine if the patient meets the inclusion criteria before providing the patient with the Participant Information Form (PIF) and obtaining contact details (telephone/mobile number and email address). Screening is based on the medical and demographic information obtained before 32 weeks gestation. From medical records and this contact, the midwife determines if the patient meets the inclusion criteria for the trial.

The contact details of pregnant individuals who meet the eligibility criteria and are interested in participating in the trial are provided to an ACE Study researcher. Individuals are contacted via telephone to discuss the trial and given an opportunity to ask questions before consenting. If verbal consent is given, the electronic consent form is sent via email (see the online Supplemental Material). Alternatively, pregnant individuals may be recruited in the antenatal care waiting room by an ACE Study researcher or at antenatal education group classes conducted at the hospital sites. The researcher conducts the eligibility screen, collects contact details from the patient, and provides the PIF.

**Figure 2.**
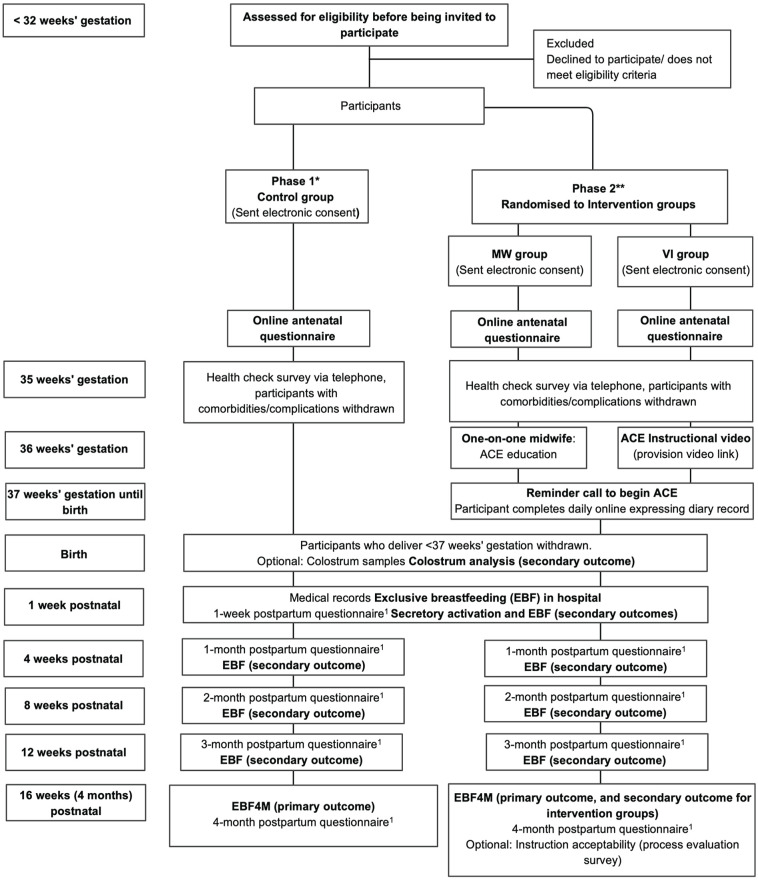
The ACE Study Flowchart. *Note.* EBF = Exclusive breastfeeding; EBF4M = Exclusive breastfeeding a 4 months. *Phase 1 control participants complete a 4-month observation period before recruitment and randomization of intervention participants. **Intervention phase. ^1^Telephone-administered or completed online by participants who cannot complete via telephone.

In addition to our in-person recruitment methods, study posters are displayed at trial sites that contain an overview of the trial and ask pregnant individuals to complete an online expression of interest survey (EOI) if they are interested in participating. The study posters contain a quick response code that links to the EOI survey. The EOI containing a link to the PIF is provided for pregnant individuals to read before completing the EOI survey. After the EOI is completed, a researcher will contact the pregnant individual via telephone and provide the individual with an opportunity to ask questions before gaining verbal consent and sending out the electronic consent form. The flow of participants through the trial from recruitment to completion is shown in Figure 2.

### Sample Size

The research team has used a control group estimate for the primary outcome, EBF4M, of 27% based on the Australian National Infant Feeding Survey (Australian Institute of Health and Welfare, 2011). This represented the best available, nationally representative breastfeeding data at the time of study design.

The research team hypothesized that an EBF4M rate of 45% will be achieved in both intervention groups. An alpha of 0.05 has been selected for the sample size calculations as (1) the tests are one-sided and (2) there are two primary hypothesis tests to be carried out. An *n* of 271 per group will be required to have over 80% power to detect a difference between the MW and VI groups, independently, as well as the control group. This will demonstrate superiority by a margin of at least 5% based on a difference in proportions using the normal approximation with an intraclass correlation of 0.01. With this *n* (271) in both the MW and VI groups, and assuming a rate of 45% will be observed for EBF4M within both intervention groups, the trial will have 80% power to demonstrate non-inferiority with a lower equivalence rate of 32%; based on the difference in proportions using the normal approximation. This lower equivalence rate was deemed acceptable as it represents the same increase over the control group as the primary analysis (i.e., 5%). Data will be analyzed based on the principles of intention to treat and per-protocol analysis, with non-inferiority concluded if both analyses produce the same results (Angeli et al., 2020; Tripepi et al., 2020). A loss to follow-up rate of 15% is anticipated; therefore, the research team estimates that 315 pregnant individuals will be recruited into each group to achieve sufficient follow-up data for analysis. Rolling recruitment across multiple hospital sites will be required to achieve the recruitment target of 945 participants.

## Data Collection and Monitoring

### Antenatal Period

All participants receive a health check via telephone between 35 and 36 weeks gestation. An ACE Study researcher administers the short health check survey to determine if a participant has developed any health conditions during pregnancy that are part of the exclusion criteria. After completion of the health check survey, participants in the intervention groups receive the ACE intervention between 36 and 37 weeks gestation. For participants in the VI group, the ACE instruction is illustrated in the ACE video where information is delivered by an IBCLC. VI group participants receive the link to the video via email. The study midwives/IBCLCs delivering the one-on-one instruction are provided with a written overview of the relevant ACE information to discuss with participants prior to conducting an instruction session. Study midwives/IBCLCs are provided with a checklist to complete during each instruction session to ensure all teaching points are covered, and the ACE technique has been demonstrated with either a knitted breast or hands-on assistance and practice (if the participant agrees). The checklist is available in the online Supplemental Material.

#### Antenatal Colostrum Expression Instruction

Intervention group participants are instructed to commence hand expressing from 37 weeks gestation, twice a day for 3–5 min per breast (maximum 10 min), using the accepted breast expression technique detailed below (Australian Breastfeeding Association, 2023). Participants are instructed to begin by stroking the breast towards the nipple and gentle breast massage to help stimulate the milk ejection reflex. Further instruction is given to make a “C” shape with the thumb and index finger and place approximately 3–4 cm on either side of the nipple, pressing in towards the chest and then pressing the fingers together and gently rolling fingers forward, being careful not to squeeze or pinch the nipple. Participants are advised to repeat the process to build up a rhythm, rotate the position of the fingers and thumb around the areola, and repeat the expressing technique to stimulate and remove colostrum from different parts of the breast. Participants are advised that small drops of colostrum may glisten on the nipple and can be collected using a sterile syringe. Participants are informed colostrum may not bead on the nipple and are reassured that this is not an indicator of potential breastfeeding ability or postpartum milk production.

Participants are provided with an intervention pack that contains a one-page handout outlining key points to remember when practicing ACE, 15 × 1 ml syringes to collect colostrum, and a cooler bag. Intervention packs can be collected from antenatal clinics at trial sites or from the midwife/IBCLC providing the one-on-one instruction. Participants are instructed to use one or more syringes for colostrum collection per day and to keep the syringes in the fridge between uses. Participants are informed that the syringes should be labelled with the date of collection, placed in a zip lock bag, and stored in their home freezer at the end of each day. Participants are provided with a bag labeled with their name to transport the colostrum syringes to the hospital at the time of birth and advised to transport the colostrum in the provided cooler bag with a freezer block. At the trial sites, there is an ACE Study box in the freezers on the maternity wards for participants to store their antenatal colostrum. After birth, the antenatally expressed colostrum can be used to supplement breastfeeding if required. If breastfeeding is going well, expressed colostrum may not be required. If colostrum syringes are not required at birth, they can be stored for 3 months in the freezer section of a refrigerator with a separate door, or 6–12 months in a deep freezer. If thawed, colostrum should be used within 4 hours if kept at room temperature, or within 24 hours if kept in the refrigerator (Australian Breastfeeding Association, 2022).

If COVID-19-related issues mean that in-person consultations are not possible, one-on-one sessions with a midwife/IBCLC may take place online. This will be noted in the data collection and when reporting findings.

Participants in the MW and VI groups are provided with direct access to an electronic milk expressing diary via Research Electronic Data Capture (REDCap; Harris et al., 2019). The diary can be accessed by email link sent daily to participants and is used to record the date they start ACE, the time they express, the volume of colostrum expressed per day, and reasons for not expressing if that occurs. The milk expressing diary has a section for additional comments where participants are encouraged to record their feelings about expressing (feelings about obtaining colostrum or not), the number of times they watch the video at home (if in the VI group), and the absence or presence of uterine contractions.

During pregnancy, participants complete the mothers’ antenatal questionnaire (MAQ) online, which is used to collect demographic information, maternal health information, breastfeeding intentions, maternal attitudes towards infant feeding, and breastfeeding self-efficacy.

### Medical Data

With consent, participants’ medical and obstetric outcome data, infant feeding method at first feed, the administration of any human milk substitutes in the hospital, and infant feeding method at hospital discharge are obtained from medical records by a research assistant.

### Postnatal Follow Up

Postnatal questionnaires are administered via telephone by a research assistant at 1, 4, 8, 12, and 16 weeks postpartum to assess infant feeding methods and/or reasons for ceasing breastfeeding. Definitions of infant feeding methods used for the ACE Study are described in Table 2. Participants’ contact points and assessments are shown on the participant timeline in Table 3.

**Table 2.** Definitions of Infant Feeding Methods Used for the ACE Study.^1^

Feeding Method	Infant Receives	Infant May Also Receive	Infant Does Not Receive
Exclusive breastfeeding	Human milk (including expressed human milk)	Oral hydration solutions, drops, syrups (vitamins, minerals, medicines)	Anything else (including water and herbal fluids)
Breastfeeding fully	Human milk (including expressed human milk)	Occasional liquids (water, water-based drinks, and juice). Oral hydration solutions, drops, syrups (vitamins, minerals, medicines)	Anything else (in particular, human milk substitutes, e.g., formula)
Combination (mixed) feeding	Human milk (including expressed human milk) and human milk substitutes	Occasional liquids (water, water-based drinks, and juice). Oral hydration solutions, drops, syrups (vitamins, minerals, medicines)	Anything else
Human milk substitute feeding	Human milk substitutes (bottle-fed)	Occasional liquids (water, water-based drinks, and juice). Oral hydration solutions, drops, syrups (vitamins, minerals, medicines)	Anything else
Other	Not specified. May include human milk substitutes (tube feeding)	Anything else	

*Note*. ACE = antenatal colostrum expression.

1 Based on the WHO Indicators for assessing infant and young child feeding practices: Definitions and measurement methods (2021).

**Table 3.** The ACE Study Participant Timeline.

Timepoint	Study Period									
	Enrollment	Allocation	Post-allocation							Close-out
	*-t_1_*	0	*t_1_*	*t_2_*	*t_3_*	t_4_	*t_5_*	*t_6_*	*t_7_*	*t_x_*
Enrollment										
Eligibility screen	X									
Informed consent	X									
Allocation		X								
Intervention										
MW				X						
VI				X						
Controls										
Assessments										
Demographics			X							
IIFAS			X							
BSES-SF			X				X			
Health check				X						
Colostrum sample					X					
Secretory activation						X				
Medical records						X				
Exclusive BF										
Process evaluation										X

*Note.* ACE = antenatal colostrum expression; MW = midwife; VI = video instruction; PASS = Perinatal Anxiety Screening Scale; EPDS = Edinburgh Perinatal/Postnatal Depression Scale; IIFAS = Iowa Infant Feeding Attitudes Scale; BSES-SF = Breastfeeding Self-Efficacy Short-Form; BF = breastfeeding. *-t_1_* = Before 32 weeks gestation; 0 = Before 34 weeks gestation; *t_1_* = 32–34 weeks gestation; *t_2_* = 35–36 weeks gestation; *t_3_* = Birth of baby; t_4_ 1-week postpartum; *t_5_* = 4-weeks postpartum; *t_6_* = 8-weeks postpartum; *t_7_* = 12-weeks postpartum; *t_x_* = 16-weeks postpartum.

### Colostrum Samples

The research team will conduct exploratory work in a subgroup of participants in the trial who are invited to provide colostrum samples for this optional, opt-in part of the trial. This data will help determine whether expressing colostrum in pregnancy has any effect on colostrum composition after birth. The research team intends to analyze post-birth colostrum samples from 35 individuals in the control group and 35 individuals from each of the intervention groups (total 70 intervention participants). This subset of participants is invited to provide two postnatal colostrum samples of no more than 1 ml each, within the first 24 hours postpartum (see the online Supplemental Material).

### Process Evaluation

A subset of participants will be invited to participate in the process evaluation which is nested within the trial to ascertain participants’ views on the acceptability of the ACE instruction and education received. This will inform future use of the ACE instructional video for teaching hand expression in pregnancy. The process evaluation will be used to understand the process that took place during participation in the ACE Study and to understand participants’ thoughts and views around expressing colostrum in pregnancy. Data will be collected using a set of standardized questions with Likert scale responses, as well as open-ended questions. The short survey will be added to the end of the 4-month post-birth questionnaire and be administered either over the telephone or as an online survey. If done over the telephone, the researcher will ask the participant if the conversation can be voice recorded to assist in capturing all relevant data when typing in responses. The recording will be deleted once the survey has been filled out by the researcher. Participants can decline to be voice recorded and still complete the survey over the telephone. Recording will be conducted using Teams if the participant provides verbal consent for this. Teams will generate a transcript of the conversation as well as an audio recording. The recording and transcription will be deleted after the data has been transferred to the survey and it is complete. The process evaluation survey will take approximately 5–10 min to complete.

Quantitative data will be analyzed using Qualtrics. Open-ended questions will be analyzed thematically and reported using the consolidated criteria for reporting qualitative research (COREQ) checklist. To ensure anonymity, data will de-identified and pseudonyms will be used to maintain confidentiality.

### Current Trial Status

The trial is ongoing and in the data collection phase. Recruitment began in September 2019, with an anticipated finish date of end. The last recruited participants are expected to reach the primary endpoint of 4 months postpartum in April 2025. Completion of data analysis is anticipated by the end of 2025.

### Informed Consent

The electronic consent form must be completed and signed before the participant is enrolled in the trial (see the online Supplemental Material). Participants who agree to donate samples of colostrum for analysis must complete an additional consent form to release the samples from the hospital sites to the trial.

### Data Management

Participants are informed before enrollment that their information will be kept confidential. The principal investigators, trial biostatistician, data monitoring committee (DMC) members, and the first author conducting the primary analysis will have access to the data. Personal demographic information, medical data obtained from medical files, and questionnaire data provided by participants will be reported anonymously. All data generated or analyzed during the current trial are not publicly available, although other investigators may request access to the dataset if a formal request describing their plans is approved by the principal investigators and the relevant ethics approval is in place.

Research records from the ACE Study will be retained for a minimum of 25 years from the date of publication or conclusion of the trial, whichever is later. Hard copies of eligibility screening forms and any other sensitive data are securely stored in a locked cabinet in an access-controlled area in the School of Medical and Health Sciences at ECU, Joondalup Campus. Only the ACE Study investigators have access to these files.

All data is managed using REDCap, an electronic data capture tool, hosted at Telethon Kids Institute. REDCap is a secure, password-protected, web-based software platform designed to support data capture for research studies, providing (1) an intuitive interface for validated data capture; (2) audit trails for tracking data manipulation and data export; (3) automated export procedures for seamless data exports in common statistical package formats; and (4) procedures for data integration and interoperability with external sources (Harris et al., 2019).

### Data Monitoring

The DMC members have been appointed with an external chair to provide an independent assessment of the safety and validity of the trial. The role of the DMC is to safeguard the interests of the trial participants and review accumulating data from the ongoing trial to assess (at regular intervals) the progress and integrity of the trial, the safety data, and the efficacy of the primary endpoint (Ellenberg et al., 2019) The DMC acts in an advisory role and provides recommendations to the principal investigators where applicable. A trial-specific charter has been formed with clear operating procedures, including the schedule and format of DMC meetings, how trial data will be presented at meetings, who has access to preliminary data, who will attend meetings, and a plan for the presentation of preliminary reports. DMC meetings are divided into open and closed sessions so the investigator (blinded to group allocation) with intimate knowledge about the progress of the trial, can attend to share information about the trial while maintaining the confidentiality of preliminary analysis results presented in closed sessions.

### Auditing

The investigators and their affiliated organizations will permit project-related monitoring, audits and regulatory inspections of all project materials, computers, and data sources at any time as required by the auditor, Human Research Ethics Committees, external sponsors, or institutional governance review bodies.

## Interventions

### Sequence Generation

The research team is using phased randomization (implemented via the stepped-wedge design), where an observation control group is recruited before commencing the intervention phase at each trial site. During Phase 2 at each trial site, participants have equal opportunity to be randomized into either the MW or VI groups. The randomization process is done using an online program for minimization (via QMinim) to ensure a balance of participants in intervention groups based on the chosen balancing variables. Two different balancing variables are used, maternal age (< 30, ≥ 30 years), and socioeconomic status (low, medium, high; determined by postcode and education level). The biased coin approach (*P* = .7; default in QMinim) is used to determine group allocation.

### Allocation Concealment Mechanism and Implementation

After randomization, the participant is sent the electronic consent link specific to the intervention arm to which they have been randomized. After consent has been obtained participants are notified via automated email of group allocation and provided further instructions relevant to the intervention to which they have been allocated.

### Blinding

Due to the nature of the intervention, neither the research midwife nor participants can be blinded to group allocation; however, the biostatistician and the investigators will be blinded to group allocation during analysis. To reduce the risk of bias, the researcher collecting data is blinded to group allocation during the intervention phase of the trial. This researcher will remain blinded to group allocation when collecting data, performing data analysis, assessing outcomes, and drafting manuscripts. Participant identifying information and group allocations will be stored in a different database to the other study-specific data fields, with records linked via a study-specific identification number. The identifying data is password-protected and only accessible to the researchers collecting the follow-up data. It is necessary during the follow-up telephone calls to ensure the correct person has been contacted and is providing the data required.

### Adherence to the Intervention

Participants in the MW and VI groups are contacted by a researcher at 37 weeks gestation via telephone to remind them to commence ACE. Compliance is monitored by viewing participants’ daily records on the expressing diary. If a participant has not completed the expressing diary record for 3 consecutive days, a reminder email is sent on the 4th day. If there is no activity on the expressing diary for a further 3 days a researcher contacts the participant via telephone. If the participant cannot be reached via telephone, a reminder email is sent to the participant.

### Participant Retention

Research assistants will make every reasonable effort to follow participants for the entire study period. The research team has developed standard operating procedures to promote participant retention and complete follow-up; researchers attempt to contact participants up to 10 times via telephone and email for a period of 4 weeks before the participant is deemed lost to follow-up.

### Safety Considerations

While ACE is considered safe according to the protocol outlined here, as a precaution participants in the intervention groups are instructed to stop ACE if they experience prolonged or frequent uterine contractions, vaginal bleeding, or feel unwell, and to notify their healthcare provider before continuing ACE. Participants are advised to contact their relevant hospital if they experience reduced fetal movements, and cease expressing until reviewed. Intervention group participants are monitored for any risks based on the participant’s activity in the expressing diary.

### Harms

If a participant reports a notable adverse event, like physical harm to their pregnancy possibly resulting from ACE, they will be withdrawn from the trial and be advised to seek immediate medical attention. In addition, if any participant reports any emotional upset or distress from being unable to express colostrum in pregnancy, they will be contacted by a researcher. The researcher will reassure the participant that not all individuals can collect colostrum in pregnancy, and this does not predict breastfeeding success. If the participant continues to express feelings of distress they will be withdrawn from the trial and advised to seek medical or therapeutic support from their preferred practitioner. The ACE Study researchers will immediately report any serious or unexpected adverse events on participants to the HREC at ECU.

## Outcomes/Measurement

Details of the primary and secondary outcome measures are provided in Table 4. In addition to recording data related to the primary and secondary outcomes, infant admission to the special care nursery (SCN), gestational age at birth, the post-term induction rate and time from starting ACE (obtained from expressing diaries) until going into labor will be recorded for MW and VI group participants to determine if ACE influences these additional outcomes.

**Table 4.** Details of the Outcome Measures Used in the ACE Study.

Outcome Measure	Measurement	Details of Measure
Primary outcome		
Exclusive breastfeeding at 4 months postpartum (EBF4M)	4-month postpartum questionnaire	Difference, between the control group and intervention groups combined (MW and VI), in the proportion of infants EBF4M
Secondary outcomes		
Exclusive breastfeeding during the initial hospital stay	Use of human milk substitutes in the hospital (medical records), 1- week postpartum questionnaire	Difference, between the control group and the intervention groups (MW and VI), in the proportion of infants exclusively breastfed in hospital
Exclusive breastfeeding at 1, 4, 8, 12 and 16 weeks postpartum	Postpartum questionnaires	Difference, between the two (MW and VI) groups, in the proportion of infants exclusively breastfed at 1, 4, 8, 12 and 16 weeks
Secretory activation (SA)	1- week postpartum questionnaire	Difference, between the control group and intervention groups (MW and VI), in the proportion of mothers who have SA at ≤ 1 day, 2-3 days, or >3 days
Colostrum composition after birth	Colostrum analysis: Macronutrients, EGF, IgA, soluble CD14, Lactoferrin and TGF	Difference, between the control group and intervention groups (MW and VI), in post-birth colostrum composition *(optional component for participants who opt-in only)*
Other outcomes		
Maternal attitudes to infant feeding	Iowa Infant Attitude Scale (IIFAS)	Difference, between the control group and intervention groups combined (MW and VI), scores on the IIFAS
Breastfeeding self-efficacy	Breastfeeding Self-Efficacy-Short-Form (BSES-SF)	Difference, between the control group and intervention groups combined (MW and VI), in BSES-SF scores
Post-term induction rate	Medical data	Difference, between the control group and intervention groups combined (MW and VI) in post-term induction rate
Time from starting ACE (obtained from expressing diaries) until labor	Expressing diaries/medical data	Difference, between the control group and intervention groups combined (MW and VI) in time from starting ACE until labor
Gestational age at birth	Medical data	Difference, between the control group and intervention groups combined (MW and VI) in gestational age at birth
Infant admission to SCN	Medical data	Difference, between the control group and intervention groups combined (MW and VI) in the proportion of infants admitted to the SCN

*Note*. ACE = antenatal colostrum expression; MW = midwife; VI = video instruction; SA = secretory activation; SCN = special care nursery; IgA = Immunoglobulin A; EGF = epidermal growth factor; TGF = transforming growth factor; MAQ = mothers’ antenatal questionnaire; IIFAS = Iowa Infant Feeding Attitudes Scale; BSES-SF = breastfeeding self-efficacy – short form.

Potential confounders identified a priori include maternal education, BMI, smoking, living in cities (Moss et al., 2020), maternal attitudes towards breastfeeding (Cox et al., 2015), and paternal breastfeeding support (Davidson & Ollerton, 2020).

### Assessment Instruments

The Iowa Infant Feeding Attitudes Scale (IIFAS), developed by Mora et al. (1999), is a reliable and valid tool used to determine maternal attitudes toward infant feeding and is included in the MAQ. The validated Breastfeeding Self-Efficacy Scale developed by Dennis and Faux (1999), is an instrument used to determine breastfeeding confidence. The shorter version, the Breastfeeding Self-Efficacy Scale-Short Form (BSES-SF; Dennis, 2003) is included in the MAQ.

Maternal secretory activation status can be assessed by maternal self-reporting of symptoms of secretory activation using the Assessment of the Onset of Lactogenesis II originally developed by Chapman and Pérez-Escamilla (2000). Maternal self-reporting of secretory activation has been shown to be a reliable indicator of secretory activation when compared with the gold standard method of test weighing an infant before and after breastfeeding to ascertain secretory activation (Rocha et al., 2020).

The ACE Study uses a version of the two-question Assessment of Onset of Lactogenesis II adapted by Demirci et al. (2023) for use in their pilot study to determine the feasibility and potential benefits of ACE in a small sample of United States mothers. Demirci et al. determined time of secretory activation by asking participants at 1–2 weeks postpartum: “How long did it take your milk to "come in” after your baby was born (i.e., when did you notice a big increase in the amount of milk)?” Demirci et al. adapted the assessment to minimize the cumulative participant survey burden in the early postpartum period. Answer options were also adapted from a by-hour recall to a by-day recall as Demirci et al. anticipated it would be difficult for participants to recall details 1–2 weeks after birth. In the ACE Study participants are asked at the 1-week postpartum questionnaire: “How long did it take your milk to “come in” after your baby was born (i.e., when did you notice a big increase in the amount of milk and a feeling of breast fullness)?” Answers are recorded to the nearest day, that is, “1 day or less,” “2 days,” “3 days,” “4 days,” “more than 4 days,” “my milk never came in,” “I don’t remember when my milk came in.” Mothers who report that their milk came in ≤ 1 day will be considered to have early secretory activation, milk onset at 2 days, or 3 days postpartum will be considered normal secretory activation, and onset at 4 days, or more than 4 days postpartum will be considered delayed secretory activation (> 72 hr postpartum). All participants repeat the BSES-SF at 4 weeks postpartum.

## Planned Data Analysis

Data will be analyzed using SPSS (Version 29) or R. The primary outcome variable is EBF4M postpartum (yes or no). The secondary outcome variables are any or exclusive breastfeeding (at 1/4/8/12/16 weeks postpartum—analyzed separately), supplementation with a human milk substitute in the hospital (yes or no), time of secretory activation (≤ 1 day, 2–3 days, or > 3 days postpartum) which are all dichotomous. Other outcome variables are infant admission to SCN (yes or no; dichotomous) and gestational age at birth (continuous). Any or exclusive breastfeeding at 1, 4, 8, 12, and 16 weeks postpartum, use of human milk substitutes in hospital, infant admission to SCN (dichotomous outcomes), and time of secretory activation (nominal outcome) will be assessed (and reported) both using unadjusted analysis (a basic comparison of proportions) and using an adjusted analysis (mixed-effects logistic regression, adjusted for maternal age, socioeconomic status, mode of delivery and potentially other hypothesized confounders). Gestational age at birth (continuous outcome) will similarly be assessed (and reported) both using unadjusted analysis (Student’s *t* test) and adjusted analysis (mixed effects linear regression, if modeling assumptions are met adjusted for maternal age, socioeconomic status, and potentially other confounders). The mixed effects models will incorporate a site variable both as a fixed effect, addressing any inter-site differences, and as a random effect, accounting for any inherent within-site correlation in the data resulting from the clustered design. The decision to include variables as confounders for adjustments in the models is based on both hypothesized and established (from related literature) relationships with the outcomes and variables of interest. An analysis of variance (ANOVA) test will be used to compare two models, one within and one without a site, by intervention interaction variable. If the model with the interaction term is deemed to be a better fit, then only pre-specified subgroup analysis will occur, which is a per-site analysis using the same models as described above. Missing data will be reported (as count and percentage) by variable and group. If problematic, multiple imputation (by chained equations) will be used to impute missing values, with reported effect estimates then calculated by pooling across models run on imputed datasets. A level of significance of *p* < 0.05 will be used, and the research team will use confidence intervals to give context to outcome variability.

### Colostrum Analysis

Colostrum samples will be analyzed at the Larsson Rosenquist Centre of Research for Immunology and Breastfeeding, University of Western Australia/Telethon Kids Institute, using indirect enzyme-linked immunosorbent assay (ELISA) for detection of IgA, TGF-beta 1, soluble CD14, Lactoferrin and EGF. Macronutrients (lactose, protein, and fat) will be measured by classical methods. Information regarding sample collection, transportation, and storage of biological specimens for analysis in the current trial can be found in the online Supplemental Material.

## Discussion

While there has been an increasing interest in ACE, this is the first comparison of different methods to deliver ACE education and instruction. Nulliparous individuals generally have lower rates of breastfeeding (Moss et al., 2020) and are more likely to have delayed secretory activation than multiparous individuals, which provides good justification for targeting this group. The results of this research will provide proof of concept evidence for the effect of ACE education in the weeks before birth on breastfeeding outcomes. If effective, improving breastfeeding rates through ACE can ultimately affect the long-term health outcomes of birthing parents and infants. The generalizability of the research findings may extend to all gravid individuals intending to breastfeed. The ACE video is advantageous as it is a cost-effective method to teach ACE, and a link to the video can be easily disseminated through current antenatal care. Similarly, should additional variants of COVID-19 or future public health emergencies cause future restrictions on accessing antenatal care, video-based ACE education might be preferable.

ACE may influence the time until secretory activation occurs. An infant requires small volumes of colostrum (approximately mean 29 ml; *SD* 24 ml) over a 24-hour period in the days after birth until secretory activation occurs (Perrella et al., 2021). Colostrum is nutrient-dense, rich in antibodies, and contains high levels of secretory Immunoglobulin A (IgA; Yang et al., 2018), which plays an important role in protecting infants from infection and providing immunity (Colonetti et al., 2022) due to immature immune systems present in the infant at birth. There is currently inadequate research on the potential effect of early secretory activation on infant health. This trial will identify if ACE influences the time to secretory activation. It will also investigate whether ACE influences postnatal colostrum composition.

This trial utilizes a non-contact approach for data collection, using telephone or online administered questionnaires to collect data. This is particularly important during the current global COVID-19 pandemic, and also convenient during the postpartum period.

### Limitations

There may be some limitations in the design of this trial; however, it is expected that the limitations will affect all three groups equally. First, because the study will be conducted only in English, it will not include non-English speaking participants. This is due to the potential language barrier that may impede the accuracy of communicating the instructions for ACE. Additionally, if participants are unable to fully understand the questions being asked in the postnatal follow-up questionnaires, this will impede the accuracy of the results. Second, there is a risk for recall bias (unintentional and intentional responder bias) resulting from possible sleep deprivation coupled with having a newborn and potential stigma/shame around the inability to breastfeed. Third, participants in the intervention groups are unable to be masked to group allocation due to the nature of the intervention.

### Protocol Amendments

If changes are made to the trial protocol (eligibility criteria, outcomes, analysis) the clinical trial registry (ANZCTR) will be updated and stakeholders will be notified via email.

## Supplemental Material

sj-docx-1-jhl-10.1177_08903344231215074 – Supplemental material for Study Protocol for a Stepped-Wedge Cluster (Nested) Randomized Controlled Trial of Antenatal Colostrum Expression (ACE) Instruction in First-Time Mothers: The ACE Study Click here for additional data file. Supplemental material, sj-docx-1-jhl-10.1177_08903344231215074 for Study Protocol for a Stepped-Wedge Cluster (Nested) Randomized Controlled Trial of Antenatal Colostrum Expression (ACE) Instruction in First-Time Mothers: The ACE Study by Cassandra Cuffe, Roslyn Giglia, Matthew N. Cooper, Julie Hill, Desiree Silva, Anita M. Moorhead, Valerie Verhasselt, Joshua R. Lewis, Deborah Ireson, Jill R. Demirci, Talea Cotte, Kathryn Webb, Frances Patey and Therese A. O’Sullivan in Journal of Human Lactation

sj-docx-2-jhl-10.1177_08903344231215074 – Supplemental material for Study Protocol for a Stepped-Wedge Cluster (Nested) Randomized Controlled Trial of Antenatal Colostrum Expression (ACE) Instruction in First-Time Mothers: The ACE Study Click here for additional data file. Supplemental material, sj-docx-2-jhl-10.1177_08903344231215074 for Study Protocol for a Stepped-Wedge Cluster (Nested) Randomized Controlled Trial of Antenatal Colostrum Expression (ACE) Instruction in First-Time Mothers: The ACE Study by Cassandra Cuffe, Roslyn Giglia, Matthew N. Cooper, Julie Hill, Desiree Silva, Anita M. Moorhead, Valerie Verhasselt, Joshua R. Lewis, Deborah Ireson, Jill R. Demirci, Talea Cotte, Kathryn Webb, Frances Patey and Therese A. O’Sullivan in Journal of Human Lactation

sj-docx-3-jhl-10.1177_08903344231215074 – Supplemental material for Study Protocol for a Stepped-Wedge Cluster (Nested) Randomized Controlled Trial of Antenatal Colostrum Expression (ACE) Instruction in First-Time Mothers: The ACE Study Click here for additional data file. Supplemental material, sj-docx-3-jhl-10.1177_08903344231215074 for Study Protocol for a Stepped-Wedge Cluster (Nested) Randomized Controlled Trial of Antenatal Colostrum Expression (ACE) Instruction in First-Time Mothers: The ACE Study by Cassandra Cuffe, Roslyn Giglia, Matthew N. Cooper, Julie Hill, Desiree Silva, Anita M. Moorhead, Valerie Verhasselt, Joshua R. Lewis, Deborah Ireson, Jill R. Demirci, Talea Cotte, Kathryn Webb, Frances Patey and Therese A. O’Sullivan in Journal of Human Lactation

sj-pdf-4-jhl-10.1177_08903344231215074 – Supplemental material for Study Protocol for a Stepped-Wedge Cluster (Nested) Randomized Controlled Trial of Antenatal Colostrum Expression (ACE) Instruction in First-Time Mothers: The ACE Study Click here for additional data file. Supplemental material, sj-pdf-4-jhl-10.1177_08903344231215074 for Study Protocol for a Stepped-Wedge Cluster (Nested) Randomized Controlled Trial of Antenatal Colostrum Expression (ACE) Instruction in First-Time Mothers: The ACE Study by Cassandra Cuffe, Roslyn Giglia, Matthew N. Cooper, Julie Hill, Desiree Silva, Anita M. Moorhead, Valerie Verhasselt, Joshua R. Lewis, Deborah Ireson, Jill R. Demirci, Talea Cotte, Kathryn Webb, Frances Patey and Therese A. O’Sullivan in Journal of Human Lactation

sj-pdf-5-jhl-10.1177_08903344231215074 – Supplemental material for Study Protocol for a Stepped-Wedge Cluster (Nested) Randomized Controlled Trial of Antenatal Colostrum Expression (ACE) Instruction in First-Time Mothers: The ACE Study Click here for additional data file. Supplemental material, sj-pdf-5-jhl-10.1177_08903344231215074 for Study Protocol for a Stepped-Wedge Cluster (Nested) Randomized Controlled Trial of Antenatal Colostrum Expression (ACE) Instruction in First-Time Mothers: The ACE Study by Cassandra Cuffe, Roslyn Giglia, Matthew N. Cooper, Julie Hill, Desiree Silva, Anita M. Moorhead, Valerie Verhasselt, Joshua R. Lewis, Deborah Ireson, Jill R. Demirci, Talea Cotte, Kathryn Webb, Frances Patey and Therese A. O’Sullivan in Journal of Human Lactation

sj-pdf-6-jhl-10.1177_08903344231215074 – Supplemental material for Study Protocol for a Stepped-Wedge Cluster (Nested) Randomized Controlled Trial of Antenatal Colostrum Expression (ACE) Instruction in First-Time Mothers: The ACE Study Click here for additional data file. Supplemental material, sj-pdf-6-jhl-10.1177_08903344231215074 for Study Protocol for a Stepped-Wedge Cluster (Nested) Randomized Controlled Trial of Antenatal Colostrum Expression (ACE) Instruction in First-Time Mothers: The ACE Study by Cassandra Cuffe, Roslyn Giglia, Matthew N. Cooper, Julie Hill, Desiree Silva, Anita M. Moorhead, Valerie Verhasselt, Joshua R. Lewis, Deborah Ireson, Jill R. Demirci, Talea Cotte, Kathryn Webb, Frances Patey and Therese A. O’Sullivan in Journal of Human Lactation
