# Supplementary material for: Study Protocol for a Stepped-Wedge Cluster (Nested) Randomized Controlled Trial of Antenatal Colostrum Expression (ACE) Instruction in First-Time Mothers: The ACE Study
Source: J Hum Lact. 2023 Dec 29;40(1):80–95. doi: 10.1177/08903344231215074 (PMC10799540; doi:10.1177/08903344231215074)
Supplement: sj-docx-1-jhl-10.1177_08903344231215074 – Supplemental material for Study Protocol for a Stepped-Wedge Cluster (Nested) Randomized Controlled Trial of Antenatal Colostrum Expression (ACE) Instruction in First-Time Mothers: The ACE Study [file sj-docx-1-jhl-10.1177_08903344231215074.docx]

# **Biological Specimens**

## **Sample collection**

Before birth, participants are provided with two syringes with caps, two cryotubes with caps (with participant ID already attached), instructions on how to collect the colostrum sample, and a biohazard bag with individual study ID number attached. Participants who provide colostrum samples for analysis self-collect samples in hospital during the first 24 hours after birth, one in the morning and the other in the afternoon/evening. The participant places the samples in the biohazard bag and gives the bag to a hospital midwife who places the samples into a -20^o^C freezer on the maternity ward. The participant notifies the ACE Study via text message that the samples are ready for collection, and an ACE researcher collects the samples from the hospital site within a week and transfers the samples to long-term storage.

**Transportation of biological specimens**

Samples are transported using a double containment method (cryotubes are secured in a plastic box using sticky tape and surrounded with paper towel, the container will have a biohazard label on the front and description of contents). Samples are then transported in a portable freezer to ***** ***** ********** laboratory for documentation and placement in long-term storage (-80 ^o^ C).

**Storage of biological specimens**

Colostrum samples will be stored in long-term storage for 6-12 months prior to analysis. Previous research demonstrates transferring samples of human milk from -20°C to -80°C does not have any effect on key macronutrients and immunoactive components (Ahrabi et al., 2016). Long-term storage (6 months) of human milk at -80°C compared with storage at -20°C is associated with better preservation of fat and energy (Orbach et al., 2019).

Colostrum can be stored at either -20°C or -80°C for 6 months before losing its immunological properties (Ramirez-Santana et al., 2012).

# **References**

Ahrabi, A. F., Handa, D., Codipilly, C. N., Shah, S., Williams, J. E., McGuire, M. A., Potak, D., Aharon, G. G., & Schanler, R. J. (2016). Effects of Extended Freezer Storage on the Integrity of Human Milk. *The Journal of Pediatrics*, *177*, 140-143. <https://doi.org/10.1016/j.jpeds.2016.06.024>

Orbach, R., Mandel, D., Mangel, L., Marom, R., & Lubetzky, R. (2019). The Effect of Deep Freezing on Human Milk Macronutrients Content. *Breastfeeding Medicine*, *14*(3), 172-176. <https://doi.org/10.1089/bfm.2018.0226>

Ramirez-Santana, C., Perez-Cano, F. J., Audi, C., Castell, M., Moretones, M. G., Lopez-Sabater, M. C., Castellote, C., & Franch, A. (2012). Effects of cooling and freezing storage on the stability of bioactive factors in human colostrum. *Journal of Dairy Science*, *95*(5), 2319-2325. <https://doi.org/10.3168/jds.2011-5066>
