# Supplementary material for: Study Protocol for a Stepped-Wedge Cluster (Nested) Randomized Controlled Trial of Antenatal Colostrum Expression (ACE) Instruction in First-Time Mothers: The ACE Study
Source: J Hum Lact. 2023 Dec 29;40(1):80–95. doi: 10.1177/08903344231215074 (PMC10799540; doi:10.1177/08903344231215074)
Supplement: sj-docx-2-jhl-10.1177_08903344231215074 – Supplemental material for Study Protocol for a Stepped-Wedge Cluster (Nested) Randomized Controlled Trial of Antenatal Colostrum Expression (ACE) Instruction in First-Time Mothers: The ACE Study [file sj-docx-2-jhl-10.1177_08903344231215074.docx]

**Supplementary Material**

# **The ACE Study Electronic Consent Form**
