# Supplementary material for: Study Protocol for a Stepped-Wedge Cluster (Nested) Randomized Controlled Trial of Antenatal Colostrum Expression (ACE) Instruction in First-Time Mothers: The ACE Study
Source: J Hum Lact. 2023 Dec 29;40(1):80–95. doi: 10.1177/08903344231215074 (PMC10799540; doi:10.1177/08903344231215074)
Supplement: sj-docx-3-jhl-10.1177_08903344231215074 – Supplemental material for Study Protocol for a Stepped-Wedge Cluster (Nested) Randomized Controlled Trial of Antenatal Colostrum Expression (ACE) Instruction in First-Time Mothers: The ACE Study [file sj-docx-3-jhl-10.1177_08903344231215074.docx]

# **Midwives’ Checklist**


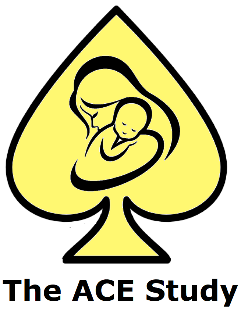


**Midwives’ Checklist for ACE Instruction Session**

Joondalup Health Campus

Date: ________________ Participant Name:___________________________

**INSTRUCTIONS:**

- Please read the *Antenatal Colostrum Expression Instructions* sheet prior to the one-on-one session.
- Complete this checklist during each session to ensure consistency of the intervention
- Discuss/demonstrate all points listed on this checklist with the participant.
- Place completed checklists in the ACE Study Tray in ******* office.

**CHECKLIST:**

Inform participant ACE should be practiced from 37 weeks’ gestation

Inform participant to wash hands before expressing, and advise how to stimulate letdown

Demonstrate hand placement and technique (C shape, press back towards chest then press fingers together and gently roll forward, rotate finger placement)

Demonstrate how to hold syringe, and collect colostrum

Discuss safe storage of colostrum: the same syringe can be used over a day, and then be placed in the freezer- make sure syringe is labelled with date the colostrum was collected

Safety instructions: Advise participant if they experience any prolonged or frequent uterine contractions, vaginal bleeding, decreased fetal movements, or feel unwell, stop hand expressing and discuss with their health care provider (GP, Midwife, Obstetrician)

Give participant ACE kit containing *Hand Expressing Instructions and syringes* to take home after the session

**Thank you very much 😊**
