# Supplementary material for: Study Protocol for a Stepped-Wedge Cluster (Nested) Randomized Controlled Trial of Antenatal Colostrum Expression (ACE) Instruction in First-Time Mothers: The ACE Study
Source: J Hum Lact. 2023 Dec 29;40(1):80–95. doi: 10.1177/08903344231215074 (PMC10799540; doi:10.1177/08903344231215074)
Supplement: sj-pdf-4-jhl-10.1177_08903344231215074 – Supplemental material for Study Protocol for a Stepped-Wedge Cluster (Nested) Randomized Controlled Trial of Antenatal Colostrum Expression (ACE) Instruction in First-Time Mothers: The ACE Study [file sj-pdf-4-jhl-10.1177_08903344231215074.pdf]

ACE 1 week post birth breastfeeding survey

Congratulations on the birth of your baby! Below are questions asking how you are feeding your baby, whether you experienced difficulties with feeding and information on your smoking status and alcohol intake.

Did your baby have ANY liquids while you were in the hospital before you gave your first breastfeed?

- ◯ Yes
- ◯ No
- ◯ Don't know

If you said 'Yes' then do you know what type of liquid baby had while in hospital before their first breastfeed?

____

How long did it take for your milk to ‘come in’ after your baby was born (i.e., when did you notice a big increase in the amount of milk and a feeling of breast fullness)?

- ◯ 1 day or less
- ◯ 2 days
- ◯ 3 days
- ◯ 4 days
- ◯ More than 4 days
- ◯ My milk never came in
- ◯ I don't remember when my milk came in

Do you remember the date and time that your milk came in?

____
(Enter in date (dd/mm/yyyy) and time in 24 hour clock)

If you cannot remember exactly when your milk came in can you think of the time of day?

Sometimes it can be helpful to think of what you were doing at the time you noticed this.

- ◯ Early morning: Between midnight - 6 am
- ◯ Morning: Between 6 am -12 noon
- ◯ Afternoon: Between 12 noon -6 pm
- ◯ Evening: Between 6 pm - midnight

If you are still waiting for your milk to 'come in' and it has been seven or more days since the birth of your baby; we recommend you contact your midwife or lactation consultant for assistance.

The next question asks what feeding method you are using. If you are unsure as to what method, you can read the definitions below to assist with answreing the below question.

Definitions of feeding for question 1a

1: Breastfeeding exclusively; your baby is only receiving breastmilk from you the mother or expressed breastmilk but no other liquids-not even water- with the exception of oral hydration solution, drop/syrups of vitamins, minerals or medicines.

2: Breastfeeding fully; your baby receives breastmilk, including expressed breastmilk but has other liquids (including water-based drinks, and fruit juice). Your baby does not receive any artificial milk. eg baby formula.

3: Combination (mixed) feeding; your baby receives both breastmilk and other fluids such as artificial milk eg, baby formula.

4: Formula feeding only (bottle fed); your baby is not receiving any breastmilk at all. Your baby is only having baby formula through the bottle.

1a: How are you feeding your baby now?

- ◯ Breastfeeding exclusively
- ◯ Breastfeeding fully (with occasional water/juice)
- ◯ Combination of breastfeeding and formula-feeding
- ◯ Formula-feeding only
- ◯ Other; please specify

if you selected 'other' in the above question then please specify what method of feeding you are using.	__________________________________
1b: Have you changed your feeding method since his/her birth?	☐ No; still feeding baby as before (GO TO Q3a)<br>☐ Yes; introduced formula<br>☐ Yes; introduced solids<br>☐ Other; please specify<br>☐ Yes, formula feeding only<br>☐ Yes; breastfeeding exclusively<br>☐ Yes; combination breastfeeding and formula feeding
if you selected 'other' in the above question then please specify what feeding change you made.	__________________________________
1c: How old was your baby when you stopped breastfeeding?	__________________________________<br>(Convert to weeks)
2a: Why did you change your feeding method?	____
2b: How old was your baby when you made this change?	__________________________________
3a: Have you had any difficulties with breastfeeding since you left hospital? Things like problems with your breasts or problems with baby feeding?	◯ Yes<br>◯ No
3b: What difficulties did you experience?<br>Select all that apply	☐ Difficult position and/or attaching my baby to the breast<br>☐ Baby has difficulties sucking<br>☐ Inverted nipples<br>☐ Not enough milk for baby<br>☐ Slow let-down of breastmilk<br>☐ Baby gets too much milk or too fast<br>☐ Breasts engorged (too full)<br>☐ Cracked or sore nipples<br>☐ Mastitis or breast infection<br>☐ Baby too tired to feed/doesn't wake up<br>☐ Baby refuses to breastfeed<br>☐ Baby not gaining enough weight<br>☐ Feeling that I am not doing very well at breastfeeding<br>☐ Breastfeeding is difficult<br>☐ Other
Specify 'OTHER' feeding difficulties you experienced. If they were not included on the above list .	__________________________________
4e: Have you heard of expressing colostrum (the early breastmilk) while you are pregnant?	◯ Yes<br>◯ No<br>◯ Unsure of what this is
4f: Have you ever expressed colostrum during this pregnancy?	◯ Yes<br>◯ No<br>◯ Unsure of what this is

When did you start expressing? (In weeks and days gestation).

____

4g: How many days did you express colostrum for during this pregnancy?

____

4h: How much colostrum were you able to collect?

(if you are unsure then provide an estimation)

____
(in millilitres)

E1: Have you fed your baby any colostrum that was collected during your pregnancy?

◯ Yes
◯ No - collected but didn't give to baby/ haven't given yet
◯ Didn't collect any

E2: In a typical 24-hour period, how often is your baby fed using the methods below?

If you use a particular feeding method less than once per day, please put “0” in the answer box.

Breastfeeding directly at the breast

____
(How many times is baby fed this way in a 24 hour period?)

Expressed breastmilk (either by hand or using a pump)

____
(How many times is baby fed this way in a 24 hour period?)

Formula

____
(How many times is baby fed this way in a 24 hour period?)

Other (please specify what other feeding method) and state how often in a 24 hour period

____

E2b: Have you expressed any breastmilk in the past week?

◯ Yes
◯ No

How many times have you expressed this past week?

____

E3. What is the main reason you express breastmilk? (tick one box)

◯ Difficulties with feeding at the breast (please specify difficulty below)
◯ Going to work/education
◯ To provide a 'top-up' feed following a breast feed
◯ To treat or prevent mastitis
◯ To increase breastmilk supply
◯ To allow others to feed the baby
◯ To store extra breastmilk (if selected, please state why below
◯ Baby is in a special care nursery/ not able to feed directly
◯ Prefer not to breastfeed
◯ Managing/collecting an oversupply
◯ Milk donation
◯ Other - please specify

Please specify what difficulties you experienced with feeding at the breast:

____

Please state why you are expressing to store extra breastmilk:

____

Please specify the main reason why you are expressing breastmilk:

____

E4. What method do you use most often to express breastmilk? (tick one box)

◯ Hand expressing
◯ Manual pump
◯ Pumping one breast at a time using a single electric pump (please specify type of pump if known below)
◯ Pumping both breasts at the same time using a double electric pump (please specify type of pump if known below)
◯ Other (please specify below)

Please specify the type of single electric pump you use if known:

____

Please specify the type of double electric pump you use if known:

____

Please specify what method you use most often:

____

5a: Since you left hospital has your baby had any drinks other than breastmilk or formula?

◯ Yes
◯ No

5b: What type of drink has your baby had?

☐ Cow's milk
☐ Water (e.g. Plain tap, filtered or mineral water)
☐ Fruit juice, fruit juice drink
☐ Ribena
☐ Cordial
☐ Soft drinks
☐ Herbal tea
☐ Other; please specify

If you selected other then can you please specify what type of drink.	______________________
6a: Since you left hospital, have you given your baby any solid foods?	◯ Yes<br>◯ No
6b: How old was your baby when you first tried him/her on solids?	______________________<br>(in weeks and days)
6c: Why did you start your baby on solids at this time?	☐ Baby was hungry/wasn't satisfied with breastmilk or formula<br>☐ Baby was old enough to have solids<br>☐ Baby reaching out for food<br>☐ To help baby sleep through the night<br>☐ Advised to start by child health nurse<br>☐ Advised to start solids by GP/specialist<br>☐ Advised to start by mother/mother-in-law<br>☐ Advised to start by other person<br>☐ Other reasons, please specify
If you selected other then please specify the reason for starting your baby on solids.	______________________
7a: Have you returned to work or study since you left hospital?	◯ No<br>◯ part-time work or study<br>◯ full-time work or study
8a: Do you smoke?	◯ Yes<br>◯ No
8b: How many cigarettes do you smoke per day on average?	______________________
9a: Do you drink alcoholic drinks?	◯ Yes<br>◯ No
9b: How many days would you drink alcohol in an average week?	______________________
9c: When during the day do you have a drink?	◯ Just before feeding your baby<br>◯ Just after feeding your baby<br>◯ In-between feeds<br>◯ At no particular time<br>◯ Just before or with the evening meal<br>◯ To coincide with feeding my baby so after feeding baby or when baby not going to wake up

Any additional comments:

If notes cannot be colleccted from medical records as planned then ask mother if she can provide inforamtion on below topics and record here in notes section ________________________________

1. Gestation/pregnancy duration in weeks and days?

2. Infant gender?

3. Birth weight in grams?

4. Did baby spend time in special care nursery and if yes how long?

5. What was baby's first feed? breast milk, formula or expressed breastmilk?

6. Did a lactation consulltant come and see you to give feeding advice while in hospital or did you request a referral to see once once discharged home?

Additional comments (Research Assistant):

e.g. call history ________________________________

Thank you very much for the information.
Either myself or one of the other ACE research assistants will be calling you again when your baby is one month old.

Thank you very much for the information on how you are feeding your baby.

You will receive your next survey to complete when your baby is one month old.
