# Supplementary material for: Study Protocol for a Stepped-Wedge Cluster (Nested) Randomized Controlled Trial of Antenatal Colostrum Expression (ACE) Instruction in First-Time Mothers: The ACE Study
Source: J Hum Lact. 2023 Dec 29;40(1):80–95. doi: 10.1177/08903344231215074 (PMC10799540; doi:10.1177/08903344231215074)
Supplement: sj-pdf-5-jhl-10.1177_08903344231215074 – Supplemental material for Study Protocol for a Stepped-Wedge Cluster (Nested) Randomized Controlled Trial of Antenatal Colostrum Expression (ACE) Instruction in First-Time Mothers: The ACE Study [file sj-pdf-5-jhl-10.1177_08903344231215074.pdf]

# Mothers' Antenatal Questionnaire

Thank you for agreeing to participate in The ACE Study. Please complete the following questionnaire which asks for your opinions and beliefs around breastfeeding and some demographic questions. It will take about 10 minutes to complete.

Your answers will be de-identified and remain strictly confidential.
When you have completed the questionnaire click submit at the end of this survey.

If you have any questions or concerns, please contact The ACE Research Team:
E: ace@ecu.edu.au
T: 08 6304 5697

The following questions will allow our research midwife to find out when you are admitted to hospital to deliver your baby.

Today's date ________________________________

How many weeks gestation are you? ________________________________

Your Estimated Due Date? ________________________________
(When is baby expected to arrive?)

Your Date of Birth ________________________________

Section 1
This section asks how you plan to feed your new baby

1 How do you intend to feed your baby when he or she is born?
- ◯ Breastfeeding only
- ◯ Mainly breastfeeding but 'topping up' with bottle feeding (infant formula)
- ◯ Mainly bottle feeding {infant formula} but also breastfeeding

2 Does the baby's father have any preference for how you feed your baby?
- ◯ Yes, he prefers breastfeeding
- ◯ Yes, he prefers bottle feeding
- ◯ He doesn't mind how I feed my baby
- ◯ Never really discussed the matter with him

**3. For each of the following statements, please indicate how much you agree or disagree by circling the number that most closely corresponded to your opinion.**

| | | Strongly agree | Agree | Neutral | Disagree | Strongly Disagree |
|---|---|---|---|---|---|---|
| 3.1 | The nutritional benefits of breast milk last only until the baby is weaned from breastmilk | ○ | ○ | ○ | ○ | ○ |
| 3.2 | Formula-feeding is more convenient than breastfeeding | ○ | ○ | ○ | ○ | ○ |
| 3.3 | Breastfeeding increases mother-infant bonding | ○ | ○ | ○ | ○ | ○ |
| 3.4 | Breastmilk is lacking in iron | ○ | ○ | ○ | ○ | ○ |
| 3.5 | Formula-fed babies are more likely to be overfed than breast-fed babies | ○ | ○ | ○ | ○ | ○ |
| 3.6 | Formula-feeding is the better choice if the mother works outside the home | ○ | ○ | ○ | ○ | ○ |
| 3.7 | Mothers who formula-feed miss one of the great joys of motherhood | ○ | ○ | ○ | ○ | ○ |
| 3.8 | Women should not breastfeed in public places such as restaurants | ○ | ○ | ○ | ○ | ○ |
| 3.9 | Babies who are fed breastmilk are healthier than babies who are fed formula | ○ | ○ | ○ | ○ | ○ |
| 3.10 | Breastfed babies are more likely to be overfed than formula-fed babies | ○ | ○ | ○ | ○ | ○ |
| 3.11 | Fathers feel left out if a mother breastfeeds | ○ | ○ | ○ | ○ | ○ |
| 3.12 | Breastmilk is the ideal food for babies | ○ | ○ | ○ | ○ | ○ |
| 3.13 | Breastmilk is more easily digested than formula | ○ | ○ | ○ | ○ | ○ |
| 3.14 | Formula is as healthy for an infant as breast milk | ○ | ○ | ○ | ○ | ○ |
| 3.15 | Breastfeeding is more convenient than formula-feeding | ○ | ○ | ○ | ○ | ○ |
| 3.16 | Breastmilk is less expensive than formula | ○ | ○ | ○ | ○ | ○ |
| 3.17 | | | | | | |

| | | | | | |
|---|---|---|---|---|---|
| A mother who occasionally drinks alcohol should not breastfeed her baby | ○ | ○ | ○ | ○ | ○ |

---

Section 2
This section asks how confident you would feel about breastfeeding when your baby is born.

**4. For each of the following statements, please select the response option that best describes how confident you will be with breastfeeding your new baby when he/she is born.**
**Please select your answer by clicking on the option that is closest to how you feel (not at all confident through to very confident). There is no right or wrong answer. The statements are useful even if you have never breastfed before or have minimal experience.**

| | | Not at all confident | Not very confident | Sometimes confident | Confident | Very confident |
|---|---|---|---|---|---|---|
| 4.1 | I will always be able to determine that my baby is getting enough milk | ○ | ○ | ○ | ○ | ○ |
| 4.2 | I will always be able to successfully cope with breastfeeding like I have with other challenging tasks | ○ | ○ | ○ | ○ | ○ |
| 4.3 | I will always be able to breastfeed my baby without using formula as a supplement | ○ | ○ | ○ | ○ | ○ |
| 4.4 | I will always be able to ensure that my baby is properly latched on for the whole feeding | ○ | ○ | ○ | ○ | ○ |
| 4.5 | I will always be able to manage the breastfeeding situation to my satisfaction | ○ | ○ | ○ | ○ | ○ |
| 4.6 | I will always be able to manage to breastfeed even if my baby is crying | ○ | ○ | ○ | ○ | ○ |
| 4.7 | I will always be able to keep wanting to breastfeed | ○ | ○ | ○ | ○ | ○ |
| 4.8 | I will always be able to comfortably breastfeed with my family members present | ○ | ○ | ○ | ○ | ○ |
| 4.9 | I will always be satisfied with my breastfeeding experience | ○ | ○ | ○ | ○ | ○ |
| 4.10 | I will always be able to deal with the fact that breastfeeding can be time consuming | ○ | ○ | ○ | ○ | ○ |
| 4.11 | I will always be able to finish feeding my baby on one breast before switching to the other breast | ○ | ○ | ○ | ○ | ○ |
| 4.12 | I will always be able to continue to breastfeed my baby for every feeding | ○ | ○ | ○ | ○ | ○ |

4.13

| | | | | | |
|---|---|---|---|---|---|
| | I will always be able to manage to keep up with my baby's breastfeeding demands | ◯ | ◯ | ◯ | ◯ | ◯ |
| 4.14 | I will always be able to tell when my baby is finished breastfeeding | ◯ | ◯ | ◯ | ◯ | ◯ |

Section 3
This section asks demographic questions of the participants who take part in the study.

5 What is your postcode?

__________________________________

5b If you don't know the post code, what suburb do you live in?

__________________________________

6 How old were you at your last birthday (years)?

__________________________________

7 What is the highest year of school you have completed (please mark only one response)?

◯ Year 12 (or equivalent)
◯ Year 11 (or equivalent)
◯ Year 10 (or equivalent)
◯ Year 9 (or equivalent)
◯ Other

7b If you selected 'Other' then please state what level of schooling you have completed up to.

__________________________________

8a What is the highest level of education you have completed?

◯ Primary school
◯ Secondary school (high school)
◯ TAFE, college
◯ University undergraduate degree
◯ University postgraduate degree
◯ Other (please specify below)

8b If you selected 'Other' then please list what form of education you have completed (eg Personal training course)

__________________________________

9 What is your usual occupation? (If currently unemployed, please give your occupation or job title prior to leaving the workforce. If you were self-employed, please give your occupation or job title e.g. hairdresser.)

__________________________________

10 What do you plan to do in the next 6 months

◯ I will still be at home with the baby
◯ work full time
◯ work part time
◯ study full time
◯ study part time
◯ work/study part time or full time from home

11 BEFORE you became pregnant, did you smoke cigarettes?

◯ Yes
◯ No

| | | |
|---|---|---|
| 11b | How many cigarettes did you smoke a day on average BEFORE you became pregnant? | __________________ |
| 12 | WHILE you are pregnant, are you smoking cigarettes? | ◯ Yes<br>◯ No |
| 12b | How many cigarettes do you smoke a day on average WHILE pregnant? | __________________ |
| 13 | How much did you weigh BEFORE you became pregnant? | __________________<br>(Answer in kilograms) |
| 14 | How tall are you? | __________________<br>(Answer in centimeters) |

15 In what country were you born?

- ◯ Afghanistan
- ◯ Albania
- ◯ Algeria
- ◯ Andorra
- ◯ Angola
- ◯ Antigua and Barbuda
- ◯ Argentina
- ◯ Armenia
- ◯ Australia
- ◯ Austria
- ◯ Azerbaijan
- ◯ The Bahamas
- ◯ Bahrain
- ◯ Bangladesh
- ◯ Barbados
- ◯ Belarus
- ◯ Belgium
- ◯ Belize
- ◯ Benin
- ◯ Bhutan
- ◯ Bolivia
- ◯ Bosnia and Herzegovina
- ◯ Botswana
- ◯ Brazil
- ◯ Brunei
- ◯ Bulgaria
- ◯ Burkina Faso
- ◯ Burundi
- ◯ Cabo Verde
- ◯ Cambodia
- ◯ Cameroon
- ◯ Canada
- ◯ Central African Republic
- ◯ Chad
- ◯ Chile
- ◯ China
- ◯ Colombia
- ◯ Comoros
- ◯ Congo Democratic Republic of the
- ◯ Costa Rica
- ◯ Côte d'Ivoire
- ◯ Croatia
- ◯ Cuba
- ◯ Cyprus
- ◯ Czech Republic
- ◯ Denmark
- ◯ Djibouti
- ◯ Dominica
- ◯ Dominican Republic
- ◯ East Timor (Timor-Leste)
- ◯ Ecuador
- ◯ Egypt
- ◯ El Salvador
- ◯ Equatorial Guinea
- ◯ Eritrea
- ◯ Estonia
- ◯ Eswatini
- ◯ Ethiopia
- ◯ Fiji
- ◯ Finland
- ◯ France
- ◯ Gabon
- ◯ The Gambia
- ◯ Georgia
- ◯ Germany
- ◯ Ghana
- ◯ Greece
- ◯ Grenada
- ◯ Guatemala

◯ Guinea
◯ Guinea-Bissau
◯ Guyana
◯ Haiti
◯ Honduras
◯ Hungary
◯ Iceland
◯ India
◯ Indonesia
◯ Iran
◯ Iraq
◯ Ireland
◯ Israel
◯ Italy
◯ Jamaica
◯ Japan
◯ Jordan
◯ Kazakhstan
◯ Kenya
◯ Kiribati
◯ Korea North
◯ Korea South
◯ Kosovo
◯ Kuwait
◯ Kyrgyzstan
◯ Laos
◯ Latvia
◯ Lebanon
◯ Lesotho
◯ Liberia
◯ Libya
◯ Liechtenstein
◯ Lithuania
◯ Luxembourg
◯ Madagascar
◯ Malawi
◯ Malaysia
◯ Maldives
◯ Mali
◯ Malta
◯ Marshall Islands
◯ Mauritania
◯ Mauritius
◯ Mexico
◯ Micronesia
◯ Moldova
◯ Monaco
◯ Mongolia
◯ Montenegro
◯ Morocco
◯ Mozambique
◯ Myanmar (Burma)
◯ Namibia
◯ Nauru
◯ Nepal
◯ Netherlands
◯ New Zealand
◯ Nicaragua
◯ Niger
◯ Nigeria
◯ North Macedonia
◯ Norway
◯ Oman
◯ Pakistan
◯ Palau
◯ Panama
◯ Papua New Guinea
◯ Paraguay
◯ Peru
◯ Philippines
◯ Poland

◯ Portugal
◯ Qatar
◯ Romania
◯ Russia
◯ Rwanda
◯ Saint Kitts and Nevis
◯ Saint Lucia
◯ Saint Vincent and the Grenadines
◯ Samoa
◯ San Marino
◯ Sao Tome and Principe
◯ Saudi Arabia
◯ Senegal
◯ Serbia
◯ Seychelles
◯ Sierra Leone
◯ Singapore
◯ Slovakia
◯ Slovenia
◯ Solomon Islands
◯ Somalia
◯ South Africa
◯ Spain
◯ Sri Lanka
◯ Sudan
◯ South Sudan
◯ Suriname
◯ Sweden
◯ Switzerland
◯ Syria
◯ Taiwan
◯ Tajikistan
◯ Tanzania
◯ Thailand
◯ Togo
◯ Tonga
◯ Trinidad and Tobago
◯ Tunisia
◯ Turkey
◯ Turkmenistan
◯ Tuvalu
◯ Uganda
◯ Ukraine
◯ United Arab Emirates
◯ United Kingdom
◯ United States of America
◯ Uruguay
◯ Uzbekistan
◯ Vanuatu
◯ Vatican City
◯ Venezuela
◯ Vietnam
◯ Yemen
◯ Zambia
◯ Zimbabwe

---

16 If you were not born in Australia, how many years have you lived in Australia? ______________________________

17 In what country was your mother born?

◯ Afghanistan
◯ Albania
◯ Algeria
◯ Andorra
◯ Angola
◯ Antigua and Barbuda
◯ Argentina
◯ Armenia
◯ Australia
◯ Austria
◯ Azerbaijan
◯ The Bahamas
◯ Bahrain
◯ Bangladesh
◯ Barbados
◯ Belarus
◯ Belgium
◯ Belize
◯ Benin
◯ Bhutan
◯ Bolivia
◯ Bosnia and Herzegovina
◯ Botswana
◯ Brazil
◯ Brunei
◯ Bulgaria
◯ Burkina Faso
◯ Burundi
◯ Cabo Verde
◯ Cambodia
◯ Cameroon
◯ Canada
◯ Central African Republic
◯ Chad
◯ Chile
◯ China
◯ Colombia
◯ Comoros
◯ Congo Democratic Republic of the
◯ Costa Rica
◯ Côte d'Ivoire
◯ Croatia
◯ Cuba
◯ Cyprus
◯ Czech Republic
◯ Denmark
◯ Djibouti
◯ Dominica
◯ Dominican Republic
◯ East Timor (Timor-Leste)
◯ Ecuador
◯ Egypt
◯ El Salvador
◯ Equatorial Guinea
◯ Eritrea
◯ Estonia
◯ Eswatini
◯ Ethiopia
◯ Fiji
◯ Finland
◯ France
◯ Gabon
◯ The Gambia
◯ Georgia
◯ Germany
◯ Ghana
◯ Greece
◯ Grenada
◯ Guatemala

◯ Guinea
◯ Guinea-Bissau
◯ Guyana
◯ Haiti
◯ Honduras
◯ Hungary
◯ Iceland
◯ India
◯ Indonesia
◯ Iran
◯ Iraq
◯ Ireland
◯ Israel
◯ Italy
◯ Jamaica
◯ Japan
◯ Jordan
◯ Kazakhstan
◯ Kenya
◯ Kiribati
◯ Korea North
◯ Korea South
◯ Kosovo
◯ Kuwait
◯ Kyrgyzstan
◯ Laos
◯ Latvia
◯ Lebanon
◯ Lesotho
◯ Liberia
◯ Libya
◯ Liechtenstein
◯ Lithuania
◯ Luxembourg
◯ Madagascar
◯ Malawi
◯ Malaysia
◯ Maldives
◯ Mali
◯ Malta
◯ Marshall Islands
◯ Mauritania
◯ Mauritius
◯ Mexico
◯ Micronesia
◯ Moldova
◯ Monaco
◯ Mongolia
◯ Montenegro
◯ Morocco
◯ Mozambique
◯ Myanmar (Burma)
◯ Namibia
◯ Nauru
◯ Nepal
◯ Netherlands
◯ New Zealand
◯ Nicaragua
◯ Niger
◯ Nigeria
◯ North Macedonia
◯ Norway
◯ Oman
◯ Pakistan
◯ Palau
◯ Panama
◯ Papua New Guinea
◯ Paraguay
◯ Peru
◯ Philippines
◯ Poland

◯ Portugal
◯ Qatar
◯ Romania
◯ Russia
◯ Rwanda
◯ Saint Kitts and Nevis
◯ Saint Lucia
◯ Saint Vincent and the Grenadines
◯ Samoa
◯ San Marino
◯ Sao Tome and Principe
◯ Saudi Arabia
◯ Senegal
◯ Serbia
◯ Seychelles
◯ Sierra Leone
◯ Singapore
◯ Slovakia
◯ Slovenia
◯ Solomon Islands
◯ Somalia
◯ South Africa
◯ Spain
◯ Sri Lanka
◯ Sudan
◯ South Sudan
◯ Suriname
◯ Sweden
◯ Switzerland
◯ Syria
◯ Taiwan
◯ Tajikistan
◯ Tanzania
◯ Thailand
◯ Togo
◯ Tonga
◯ Trinidad and Tobago
◯ Tunisia
◯ Turkey
◯ Turkmenistan
◯ Tuvalu
◯ Uganda
◯ Ukraine
◯ United Arab Emirates
◯ United Kingdom
◯ United States of America
◯ Uruguay
◯ Uzbekistan
◯ Vanuatu
◯ Vatican City
◯ Venezuela
◯ Vietnam
◯ Yemen
◯ Zambia
◯ Zimbabwe

---

18 What is your marital status?

________________________________
