# Supplementary material for: Study Protocol for a Stepped-Wedge Cluster (Nested) Randomized Controlled Trial of Antenatal Colostrum Expression (ACE) Instruction in First-Time Mothers: The ACE Study
Source: J Hum Lact. 2023 Dec 29;40(1):80–95. doi: 10.1177/08903344231215074 (PMC10799540; doi:10.1177/08903344231215074)
Supplement: sj-pdf-6-jhl-10.1177_08903344231215074 – Supplemental material for Study Protocol for a Stepped-Wedge Cluster (Nested) Randomized Controlled Trial of Antenatal Colostrum Expression (ACE) Instruction in First-Time Mothers: The ACE Study [file sj-pdf-6-jhl-10.1177_08903344231215074.pdf]

WK16 ACE Postnatal Participant Survey

Below are questions asking how you are feeding your baby at sixteen weeks (4 months) after their birth.
These questions will be similar to those asked in previous post birth surveys. It should take around 5 minutes to complete.

The first question asks what feeding method you are using. If you are unsure as to what method, you can read the below definitions to assist with asnwering the question.

Definitions of feeding for question 1a

1: Breastfeeding exclusively; your baby is only receiving breastmilk from you the mother or expressed breastmilk but no other liquids-not even water- with the exception of oral hydration solution, drop/syrups of vitamins, minerals or medicines.

2: Breastfeeding fully; your baby receives breastmilk, including expressed breastmilk but has other liquids (including water-based drinks, and fruit juice). Your baby does not receive any artificial milk. eg baby formula.

3: Combination (mixed) feeding; your baby receives both breastmilk and other fluids such as artificial milk eg, baby formula.

4: Formula feeding only (bottle fed); your baby is not receiving any breastmilk at all. Your baby is only having baby formula through the bottle.

1a: How are you feeding your baby now?	◯ Breastfeeding exclusively<br>◯ Breastfeeding fully (with occasional water and juice)<br>◯ Combination of breastfeeding and formula-feeding<br>◯ Formula-feeding only<br>◯ Other; please specify
Specify other method of feeding your baby	______________________________
1b Have you changed your feeding method since the last survey?	☐ No; still feeding baby as before (GO TO Q3a)<br>☐ Yes; introduced formula<br>☐ Yes; introduced solids<br>☐ Other; please specify<br>☐ Yes, formula feeding only<br>☐ Yes; breastfeeding exclusively<br>☐ Yes; combination breastfeeding and formula feeding
if you selected 'other' in the above question then please specify what feeding change you made.	______________________________
1c: How old was your baby when you stopped breastfeeding?	______________________________<br>(Convert to weeks)
2a: Why did you change your feeding method?	______________________________
2b: How old was your baby when you made this change?	______________________________

If you were not breastfeeding at the last survey then Go to question 5a.

3a: Have you had any difficulties with breastfeeding since the last interview? Things like problems with your breasts or problems with baby feeding?	◯ Yes<br>◯ No
3b: What difficulties were experienced?	☐ Difficult position and/or attaching my baby to the breast<br>☐ Baby has difficulties sucking<br>☐ Inverted nipples<br>☐ Not enough milk for baby<br>☐ Slow let-down of breastmilk<br>☐ Baby gets too much milk or too fast<br>☐ Breasts engorged (too full)<br>☐ Cracked or sore nipples<br>☐ Mastitis or breast infection<br>☐ Baby too tired to feed/doesn't wake up<br>☐ Baby refuses to breastfeed<br>☐ Baby not gaining enough weight<br>☐ Feeling that I am not doing very well at breastfeeding<br>☐ Breastfeeding is difficult<br>☐ Other
Specify other feeding difficulty	__________________________________
E1: Have you fed your baby any colostrum/breastmilk that was collected during your pregnancy?	◯ Yes<br>◯ No - collected but didn't give to baby/ haven't given yet<br>◯ Didn't collect any
E2: In a typical 24-hour period, how often is your baby fed using the methods below?<br><br>If you use a particular feeding method less than once per day, please put “0” in the answer box.	
Breastfeeding directly at the breast:	__________________________________<br>((How many times is baby fed this way in a 24-hour period?))
Expressed breastmilk (either by hand or using a pump):	__________________________________<br>((How many times is baby fed this way in a 24-hour period?))
Formula:	__________________________________<br>((How many times is baby fed this way in a 24-hour period?))
Other (please specify what other feeding method) and state how often in a 24 hour period	__________________________________
E2b: Have you expressed any breastmilk in the past week?	◯ Yes<br>◯ No
How many times have you expressed this past week?	__________________________________

E3. What is the main reason you express breastmilk? (tick one box)	◯ Difficulties with feeding at the breast (please specify difficulty below)<br>◯ Going to work/education<br>◯ To provide a 'top-up' feed following a breast feed<br>◯ To treat or prevent mastitis<br>◯ To increase breastmilk supply<br>◯ To allow others to feed the baby<br>◯ To store extra breastmilk (if selected, please state why below<br>◯ Baby is in a special care nursery/ not able to feed directly<br>◯ Prefer not to breastfeed<br>◯ Managing/collecting an oversupply<br>◯ Milk donation<br>◯ Other - please specify
Please specify what difficulties you experienced with feeding at the breast	______________________
Please state why you are expressing to store extra breastmilk:	______________________
Please specify the main reason why you are expressing breastmilk:	______________________
E4. What method do you use most often to express breastmilk? (tick one box)	◯ Hand expressing<br>◯ Manual pump<br>◯ Pumping one breast at a time using a single electric pump (please specify type of pump if known below)<br>◯ Pumping both breasts at the same time using a double electric pump (please specify type of pump if known below)<br>◯ Other (please specify below)
Specify the type of single electric pump you use if known:	______________________
Specify the type of double electric pump you use if known:	______________________
Please specify what method you use most often:	______________________
5a: Since the last survey has your baby had any drinks other than breastmilk or formula?	◯ Yes<br>◯ No
5b: What type of drink has your baby had?	☐ Cow's milk<br>☐ Plain tap, filtered or mineral water<br>☐ Fruit juice, fruit juice drink<br>☐ Ribena<br>☐ Cordial<br>☐ Soft drinks<br>☐ Herbal tea<br>☐ Other; please specify
Specify other type of drink	______________________

6a: Since the last survey, have you given your baby any solid foods?	◯ Yes<br>◯ No
6b: How old was your baby when you first tried him/her on solids?	____________________<br>(in weeks and days)
6c: Why did you start your baby on solids at this time?	☐ Baby was hungry/wasn't satisfied with breastmilk or formula<br>☐ Baby was old enough to have solids<br>☐ Baby reaching out for food<br>☐ To help baby sleep through the night<br>☐ Advised to start by child health nurse<br>☐ Advised to start solids by GP/specialist<br>☐ Advised to start by mother/mother-in-law<br>☐ Advised to start by other person<br>☐ Other reasons, please specify
Specify other reason for starting baby on solids	____________________
7a: Have you returned to work or study since the last survey?	◯ No<br>◯ part-time work or study<br>◯ full-time work or study
8a: Do you smoke?	◯ Yes<br>◯ No
8b: How many cigarettes do you smoke per day on average?	____________________
9a: Do you drink alcoholic drinks?	◯ Yes<br>◯ No
9b: How many days would you drink alcohol in an average week?	____________________
9c: When during the day do you have a drink?	◯ Just before feeding your baby<br>◯ Just after feeding your baby<br>◯ In-between feeds<br>◯ At no particular time<br>◯ Just before or with the evening meal<br>◯ To coincide with feeding my baby so after feeding baby or when baby not going to wake up
Additional Comments:	____________________
Research Assistant Additional Comments:	____________________

Thank you very much for the information on how you are feeding your baby.

This was the last survey you were required to complete!

Well done on making it to the end of the ACE breastfeeding study!
